# Prevalence of Fascioliasis and Associated Economic Losses in Cattle Slaughtered at Lira Municipality Abattoir in Northern Uganda

**DOI:** 10.3390/ani11030681

**Published:** 2021-03-04

**Authors:** Lawrence George Opio, Essam M. Abdelfattah, Joshua Terry, Steven Odongo, Emmanuel Okello

**Affiliations:** 1 Department of Biotechnical and Diagnostic Sciences, College of Veterinary Medicine, Animal Resources and Biosecurity, Makerere University, Kampala 7062, Uganda; lawgeorgeo@gmail.com (L.G.O.); opodongo@covab.mak.ac.ug (S.O.); 2 Veterinary Medicine Teaching and Research Center, School of Veterinary Medicine, University of California Davis, Tulare, CA 95616, USA; eabdelfattah@ucdavis.edu (E.M.A.); jdterry@ucdavis.edu (J.T.); 3 Department of Animal Hygiene, and Veterinary Management, Faculty of Veterinary Medicine, Benha University, Qalyubia 13518, Egypt; 4 Department of Population Health and Reproduction, School of Veterinary Medicine, University of California Davis, Davis, CA 95616, USA

**Keywords:** cattle, fascioliasis, prevalence, economic loss, Northern Uganda

## Abstract

**Simple Summary:**

Fascioliasis is an economically important parasitic snail-borne disease of ruminant animals including cattle, sheep and goats that has public health significance due to risk of infection transmission to humans. The disease causes growth retardation, decreased milk and meat production, and liver damage in infected animals. The current cross-sectional study was conducted at the Lira Municipal abattoir in Northern Uganda to estimate the prevalence of fascioliasis and evaluate the risk factors (region, breed, sex, and age of animals) associated with infection in slaughter cattle. Each selected carcass was examined for fascioliasis by dissecting the liver. Infested sections of the livers were trimmed off, condemned and incinerated. Out of the 216 liver samples examined, the majority (65.7%; *n* = 142) were infested with *Fasciola* spp. This study revealed that cattle aged 4 years or older were at a higher risk of fascioliasis in comparison to young cattle (0–3.5 years). The estimated monetary loss due to liver damage was 9900 UGX (2.67 USD)/infected animal. The outcome of this study underscores the need for increased awareness of the disease burden, and implementation of appropriate control measures.

**Abstract:**

Fascioliasis (liver fluke infestation) is one of the most important parasitic diseases affecting cattle, other ruminant animals and humans. Fascioliasis causes large, but usually neglected, economic losses to cattle farmers and traders. The objectives of this study were to assess the prevalence and associated risks for fascioliasis in slaughter cattle and estimate the financial losses due to liver condemnation at the Lira Municipal abattoir in Uganda. A total of 216 cattle were sampled during the study period. Animal breed and sex were determined by observing the phenotypic characteristics of the animals. Age was determined by assessing the eruption and wearing of permanent teeth. After slaughter, the liver was examined for presence of *Fasciola* spp. (liver flukes) by visual inspection, palpation, and incisions. The bile ducts and gall bladder were similarly examined for presence of mature *Fasciola* spp. The gross weight and amount of liver trimmed-off due to fluke infestation were determined. Of the 216 liver examined, 65.7% (*n* = 142) were infested with *Fasciola* spp. Cattle that were aged 4–5 years old at the time of slaughter had significantly greater odds (OR = 5.84; CI [2.79–12.22]) of being infested with *Fasciola* spp. compared to those that were younger than 3.5 years old. In contrast, cattle that had a body condition score of 3.5 or 4 had lower odds (OR= 0.42; CI [0.21–0.88] and OR = 0.22; CI [0.04–1.10]) of fascioliasis than those with a BCS of 3. Other tested variables including animal origin, breed, sex, and gross weight of the liver had no significant effect on the prevalence of fascioliasis. This study also revealed that the abattoir loses an estimated 38 million UGX annually due to condemnation of *Fasciola*-infested liver (one UGX= 0.00027 USD; July 2016). Our study showed that the prevalence of fascioliasis was high in Lira District, Uganda, which results in a large amount of liver being condemned and destroyed, leading to financial losses for affected farmers in the area. Therefore, there is a need to take the necessary preventive measures to control the disease and increase awareness among farmers and medical personnel in the area due to the zoonotic nature of fascioliasis.

## 1. Introduction

Fascioliasis, also known as liver rot, is an important parasitic infection disease of cattle caused by two main species of trematodes: *Fasciola gigantica*, and *Fasciola hepatica* [1]. In Europe, the Americas, and Oceania, only *Fasciola hepatica* is a concern, but the distributions of both *Fasciola* spp. overlap in many areas of Africa and Asia [2]. The *Lymnaea* snails living along the riverbanks are suitable intermediate hosts for *Fasciola* spp. [3]. The prevalence of fascioliasis varies between herds and regions. In 2015, a study conducted on farms from the Nile Delta region in Egypt reported a herd prevalence of 9.77% [4]. In Denmark, an increase in annual herd prevalence was reported between the years 2011 (25.6%) to 2013 (29.3%) [5]. At the animal level, several studies have reported fascioliasis prevalence ranging from 10% to 50.5% in slaughter animals from different abattoirs in South Africa [6], Ethiopia [7] and Nigeria [8,9].

Fascioliasis causes acute and chronic infections [10], and it occurs chiefly in cattle, sheep, goats, and buffalos but may affect humans and other species [1]. As meat consumption increases worldwide, there are growing concerns about meat hygiene and safety, especially given that the worldwide distribution of fascioliasis is estimated at 90% in ruminants [11]. According to Mas-Coma et al. [2,12], and Esteban et al. [13], fascioliasis has recently been shown to be a re-emerging and widespread zoonosis affecting multiple human populations, hence gaining attention as a public health issue. In addition, fascioliasis is now recognized as an emerging human disease. The World Health Organization has estimated that 2.4 million people are infected with *Fasciola* spp. and a further 180 million are at risk of infection [14]. 

Economically, worldwide losses in animal productivity due to fascioliasis were conservatively estimated at over $3.2 billion USD per annum according to 1999 publication [15]. The economic losses are categorized as direct losses, which consist of drug costs, drenches, labor, and liver condemnation at abattoirs; and indirect losses associated with decreased productivity such as reduced production, poor growth rate, increased costs for replacement stock, reduced production and quality of milk, and lower feed conversion rates in cattle [16]. Therefore, the aim of this study was to estimate the point prevalence of fascioliasis and evaluate risk factors and the direct financial loss caused by *Fasciola* spp. in slaughtered cattle in Northern Uganda.

## 2. Materials and Methods

### 2.1. Study Site

**Figure 1:**
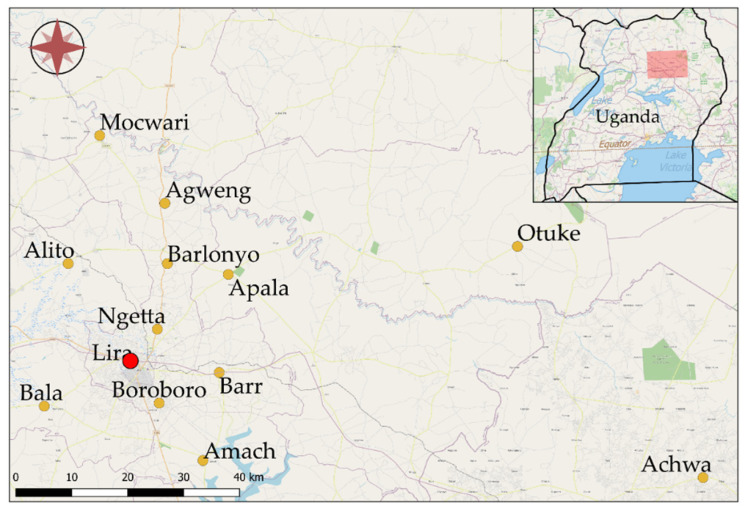
Sources of animals slaughtered at Lira Municipal abattoir in Uganda, Africa. The abattoir location is marked by the red dot. Sampling locations were reclassified into three regions: Southern (Achwa, Amach, Bala, Boroboro), Eastern (Apala, Barr, Otuke), and Northern regions (Agweng, Alito, Barlonyo, Mocwari, Ngetta).

This was a cross-sectional study conducted in July 2016 at Lira Municipal abattoir, Lira District, Northern Uganda (Figure 1). Lira Municipality is located at latitude 20′ 17′ north of the equator and longitude 32′ 56′ east of the principal meridian. The municipality consists of four sub-counties including Central division, Railways division, Ojwina division and Adyel division. Over the course of this study, the temperature ranged from 28.88 to 27.77 °C and relative humidity ranged from 8% to 12%. The wetter season lasts 7.4 months, from 1 April to 14 November, with a greater than 34% chance of a given day being a wet day. The slaughtered animals were sourced from within Lira district and nearby surrounding districts (Table 1). 

### 2.2. Sample Collection and Gross Examination

At the abattoir, information on individual animal source was recorded. Physical examination (antemortem) was performed shortly prior to slaughter in order to determine animal breed, sex, age, and body condition score. Animal breed and sex were determined by observation of phenotypic characteristics of each animal. Age of cattle was determined using a guide [17], which gauges age based on the eruptions, and wear of permanent teeth. Body condition for each cattle was estimated based on a 5-point scoring system according to Wildman et al. [18], ranging from score 1 (emaciated) to score 5 (obese). The classes of scoring used were emaciated (score 1), lean (score 2), medium (score 3 and 4) and obese (score 5). After slaughter of animals, the liver and associated bile duct was carefully examined at post-mortem (PM) for presence of liver flukes and associated pathology by visualization inspection and palpation. Further examination of the liver for liver flukes was conducted by making incisions into the bile ducts and gall bladder. The condemnation of the liver was based on the guidelines on meat inspection for developing countries [19]. According to these guidelines, liver that is infested with *Fasciola* at slaughter is only partially condemned; only the liver sections containing the *Fasciola* parasites and/with gross sclerosis condemned and trimmed off. Livers classified as totally condemned because of gross and generalized morphological abnormalities were rejected and destroyed to avoid human consumption. In cases of partial condemnation, the liver was trimmed, and the weight (in metric units) of the trimmed part was taken. All livers were weighed before and after trimming. The monetary loss was calculated based on current market price (in UGX) per kilogram as of July 2016 (one UGX = 0.00027 USD). 

### 2.3. Liver Condemnation and Associated Financial Loss

The financial loss due to liver condemnation was assessed by calculating the price value of the portion of liver trimmed due to fascioliasis infestation. One kilogram of liver on the local market cost 10,000 UGX on average. The unit monetary loss was calculated by multiplying the amount in kilograms trimmed off by the market value of liver. The total loss was calculated by multiplying the unit monetary loss by frequency. This was used to compute the estimated financial loss for the entire study period. 

### 2.4. Sample Size Calculation

The sample size was determined using the Cochran formula [20]. With the assumption of 84% expected prevalence of fasciolosis, at 95% CI and 5% level of precision, the total sample size computed was 206 cows. 

### 2.5. Data Management and Analysis

The data collected were entered into Microsoft Excel 2010 spreadsheet and exported to Stata software for analysis. Frequencies and proportions, and their standard error (SE), were computed for categorical and ordinal variables. The continuous data were checked for normality using normal quantile, and normal probability plots in Stata. Mean and SE were computed for continuous variables. Confidence intervals for proportions were calculated using the normal distribution approximation method. Data on the location of cows were reclassified into three regions: Northern regions (Agweng, Alito, Barlonyo, Mocwari, Ngetta), Southern (Achwa, Amach, Bala, Boroboro), and Eastern (Apala, Barr, Otuke). The age of cows was classified into three groups: young (0–3.5 years), middle (4–5 years), and adult cows (6–10 years). The gross liver weight data were categorized into 3 levels: (1) from 0 to 2 kg, (2) from 2.1 to 4 kg, and (3) from 4.1 to 6 kg. Logistic regression models using logit function were used to study the association between dichotomous outcome variable (*Fasciola* spp. infestation) and explanatory variables (region, breed, sex, BCS, liver gross weight, and age of animal at slaughter). Two-way interactions for potential effect modifiers were tested using significance testing (*p* < 0.05). Information theory’s Akaike’s Information Criterion (AIC) was used to build and compare competing models and the model with the lowest value was deemed as the best fit. Odds ratio (OR) and associated SE were represented in final tables. All statistics were performed using Stata 15 software (Stata Corp, College Station, TX, USA).

## 3. Results

### 3.1. Socio-Demographic Characteristics of Cattle Assessed in This Study

Characteristics of animals included this study are summarized in Table 1. Most of the animals assessed were of the small East African Zebu breed, accounting for 89.8% (*n* = 194) of the sample population, with a few animals of the Ankole breed, accounting for 10.2% (*n* = 22) of the total animals assessed (*n* = 216). The majority of the cattle slaughtered were males (74.1%, *n* = 160) while females comprised 25.9% (*n* = 56). The age distribution of the slaughtered animals was as follows: 18.5% (*n* = 40) of cattle were 3.5 years old, 37.5% (*n* = 81) were 4 years old, 20.4% (*n* = 44) were 5 years old, and 6.9% (*n* = 15) were 6 years old.

### 3.2. Prevalence of Fascioliasis in Slaughtered Cattle

Out of the 216 liver examined, 65.7% (*n* = 142) were infested with *Fasciola* spp. (Table 2). The Small East African Zebu breed had a higher prevalence of infestation (67.5%, *n* = 131) when compared to the Ankole breed (50%, *n* = 11). Between sexes, males had a higher prevalence of fasciolosis 68.8% (*n* = 110) than females 57.1% (*n* = 32). Among the various age groups, cattle aged 4–5 years old had higher fasciolosis prevalence (75.3%) compared to cows younger than 4 years (43.24%) or cows older than 6 year olds (70.58%). Additionally, cows with a BCS of 3 had higher fascioliasis prevalence (69.33%) compared to cows with a BCS of 3.5 (64.34%) or BCS of 4 (58.33%) (Table 2).

### 3.3. Risk Factors for Fasciola Infestation

Logistic regression models were used to estimate the association between *Fasciola* spp. infestation and various animal and environmental factors as predictors for *Fasciola* infestation at the time of slaughter (Table 3). Our results showed that cattle age at slaughter, and body condition score were the main factors predicting *Fasciola* spp. infestation. Cattle that were aged 4–5 years old at the time of slaughter had significant greater odds (OR = 5.84) of being infested with fascioliasis compared to those that were younger than 3.5 years old (Table 3). Furthermore, our results indicated that cattle with body condition scores of 3.5 or 4 had lower odds (OR = 0.42 and 0.22) of being infested with fascioliasis than those with a BCS of 3. Other tested variables including animal origin, breed, sex, and gross weight of the liver had no significant effect on the prevalence of fascioliasis (Table 3).

### 3.4. Financial Losses Associated with Liver Condemnation 

Financial losses due to fasciolosis were assessed by calculating the value of the liver sections trimmed due to *Fasciola* infestation. During the 2 week study period, a total of 145.7 kg of livers was trimmed from 141 slaughtered cattle that had with *Fasciola*-infested liver, which averaged to 0.99 kg/infected animal. At the time of this study (July 2016), one kilogram of liver on the local market cost 10,000 UGX or 2.7 USD on average at the time of this study; hence, the estimated monetary loss was 9900 UGX (2.67 USD)/infected animal (one UGX = 0.00027 USD; July 2016). The total estimated financial loss within the two weeks of the study period was 1.457 million UGX, equivalent to 396.41 USD, and the annual projected loss was 37.88 million UGX (10,306.66 USD). The minimum unit monetary loss per animal was 3000 UGX, while the maximum was 50,000 UGX per animal (Table S1).

## 4. Discussion

Fascioliasis is a serious infectious parasitic disease in domestic ruminant animals [3]. The prevalence and financial losses associated with liver condemnation due to fascioliasis was assessed at Lira Municipal abattoir in this study. The prevalence of fascioliasis was generally high; 65.7% of the total cattle carcasses examined were infested. This prevalence approximately agrees with findings from similar studies by Howell et al., [21], which reported a prevalence of 64.5% in a study performed on the slopes of Mount Elgon, Uganda; and with Jean-Richard et al. [22], which reported a prevalence of 68% in South Eastern Lake Chad. However, a much higher prevalence of 75% was reported by Ssimbwa et al., [23] in a study to determine the prevalence and financial losses associated with fascioliasis at Lyantonde town abattoir. A more recent study by Joan et al. [24] at Kampala City Abattoir, Central Uganda reported a prevalence of 84%. Despite several studies reporting high rates of prevalence, low prevalence was reported in several other studies as well. For example, [25] reported a low prevalence of 13.4% of *Fasciola gigantica* in Onitsha Nigeria. 

The difference in these prevalence reports could be attributed to several factors, one of which could be the season of the study. Studies performed in rainy seasons generally reported high prevalence compared to those performed in dry seasons. This could be attributed to the fact that the snail, which serves as the intermediate host, abounds in rainy season [25,26]. Another factor could be type of sample examined, whether liver or feces. A study conducted by [11] reported that detection of fascioliasis through liver examination at PM is more sensitive than coprological assay. The sensitivity of the test used could also account for this difference—for example, Nguyen et al. [27] reported a higher prevalence with ELISA test compared to fecal and liver examination. In addition, there are management systems that expose cattle to eggs. For example, animals managed under intensive management system are less likely to be infected compared to those that are managed under an extensive system. On the other hand, higher prevalence is likely to be encountered in herds receiving poor veterinary services and/or irregular deworming practices. 

This study also revealed that there was a significant association between body condition score and fascioliasis infestation. Animals with a BCS of 3 at time of slaughter were most affected by *Fasciola* spp. (69.33%). This result agrees with results obtained by Nyirenda et al. [28] that showed that the most prevalent liver fluke identified was *Fasciola gigantica* (56.1%) and it mostly affected animals with a poor body condition (71.4%). Many studies showed a positive association between fasciolosis and BCS [29,30,31,32].

Our results also showed that young cattle of 4–5 years old were highly infested with *Fasciola* spp. in comparison to cows ≥6 years. This in agreement with the previous study conducted in South Africa [33], with risk factors such as age (young: 18.2% and old: 14.1%) and sex (male: 29.1% and female: 3.2%). The results of the current study revealed that the infection rate was significantly higher in 4–5-year-old animals than in young animals. Cattle at this age frequently graze pastures and have longer exposure time, which may increase the likelihood of infection with *Fasciola metacercariae* [34]. Additionally, the low prevalence in older cattle (6–10 years) can be attributed to the high immunogenicity of the parasite, which aids in the stimulation of acquired immunity in older animals [35].

The sex of animals was not associated with the infestation rate. Similar to our results, Khan et al. (2009) found no sex-related differences in prevalence of fasciolosis [36]. However, the prevalence of fasciolosis in our study was higher in males than females. A study in Nigeria showed higher infection rates in males than females [37]. The higher prevalence in males could be attributed to several factors, including grazing disparity between both sexes, especially if the cow is pregnant, and the fact that females are mainly used for milk production and seldom raised for beef production [31,35,38]. This finding agrees with Magaji et al. [3], who reported that the disparity in susceptibility between sexes could be attributed to intrinsic factors such as genetics, physiology and immunology and extrinsic factors such as environment and management practices.

Furthermore, there was no significant association between cattle breed and infestation. However, the Small East African Zebu breed had a higher prevalence compared to the Ankole breed. This could be due to better management and husbandry practices such as zero grazing and more intensive veterinary care for the Ankole breed compared to the Zebu breed. In most cases, the Zebu is managed under extensive grazing systems which predispose them to water from swampy areas despite being presumed to be highly resistant to parasites and diseases. 

Our study also revealed abattoir losses of 1,457,000 UGX within the study period of two weeks because of liver condemnation due to *Fasciola* spp. infestation, which is equivalent to 37,882,000 UGX after a year. This financial loss is less than the financial loss reported by Joan et al. [24], 231,186,550,000 UGX, in their study at Kampala City Abattoir, Central Uganda. This could be attributed to the fact that many more cattle are slaughtered per day in the Kampala abattoir compared to the Lira municipality abattoir, probably due to a lower population of meat consumers in Lira. This financial loss is also less than the economic loss of 1.5 million USD reported in Kenya [26]. This could be attributed to the fact that many abattoirs were sampled in Kenya while only one abattoir was sampled in this study. 

These results highlight significant decreases in the household incomes of small-scale farmers, as reported elsewhere [39,40]. Therefore, effective control measures should be taken to minimize these losses through the control of the intermediate hosts. It is also important to educate the public about the significance of this zoonotic disease. Furthermore, meat inspection should be intensified in all abattoirs to monitor the prevalence of the disease and avoid humans ingesting the liver flukes in infected cattle livers.

One drawback of this study was the cross-sectional design, implemented at a single time point. Any projections of prevalence estimate for *Fasciola* infestation and associated financial losses could be affected by seasonal variations in infection rates. In addition, animal trade and movements are common within the study area, but we only recorded the last reported area of origin of the slaughter animals prior to transportation to the abattoir. The sample size calculated in this study was based on a prevalence of 84%, but our study estimated a prevalence of 65.7%.

## 5. Conclusions

Our study revealed that the prevalence of *Fasciola* spp. was generally high in Lira Municipal abattoir, Uganda. *Fasciola* spp. infestation was associated with significant financial losses for farmers in that area. Our study also revealed that there was a significant association between age of animal and body condition score and *Fasciola* spp. infestation in cattle. Continuous meat inspection should be performed since the prevalence was significantly high in the area to avoid human infection by liver flukes. Additional studies should be carried out in this area to assess the zoonotic risk and human infestation with fasciolosis.

## Figures and Tables

**Table 1:** Summary of distribution and characteristics of slaughter cattle (*n* = 214) at Lira Municipal abattoir examined for *Fasciola* spp. infestation. Physical characteristics were determined during antemortem examination, while liver samples were examined post-slaughter.

Variable	*n*	Percentage
Region		
Southern ^a^	131	54.17
Eastern ^b^	76	35.19
Northern ^c^	9	10.65
Breed		
Small East African Zebu	194	89.81
Ankole	22	10.19
Sex		
Male	160	25.93
Female	56	74.10
Age of animal at slaughter, years		
0–3.5	74	34.26
4–5	125	57.87
6–10	17	7.87
Body condition score		
3	75	34.72
3.5	129	59.72
4	12	5.56
Gross weight of liver at slaughter (Kg)		
0–2	50	23.15
2.1–4	149	68.98
4.1–6	17	7.87
*Fasciola* infestation		
Yes	142	65.74
No	74	34.26
Trimmed part of liver (Kg)		
0–1	169	78.24
1.1–2	36	16.67
2.1–5	11	5.10

^a^ Southern = Achwa, Amach, Bala, and Boroboro; ^b^ Eastern = Apala, Barr, and Otuke; ^c^ Northern = Agweng, Alito, Barlonyo, Mocwari, and Ngetta.

**Table 2:** Prevalence of fascioliasis in examined cattle (*n* = 142) assessed by region, breed and sex, age, and source of animals.

Category	*n*	Percentage
Region ^1^		
Southern	88	64.10
Eastern	48	63.15
Northern	6	82.61
Breed		
Small East African Zebu	131	67.52
Ankole	11	50
Sex		
Female	110	68.75
Male	32	57.14
Age of animal at slaughter, years		
0–3.5	32	43.24
4–5	98	78.40
6–10	12	70.58
Body condition score		
3	52	69.33
3.5	83	64.34
4	7	58.33
Gross weight of liver at slaughter, kg		
0–2	28	56
2.1–4	100	67.11
4.1–6	14	82.35

^1^ Southern = Achwa, Amach, Bala, and Boroboro; Eastern = Apala, Barr, and Otuke; Northern = Agweng, Alito, Barlonyo, Mocwari, and Ngetta.

**Table 3:** Estimated odds ratio (OR) from logistic regression model for the association between fascioliasis infestation (Yes, No) and explanatory variables (region, breed, sex, body condition score, and liver gross weight).

Variable	OR	SE	95% CI		*p*-Value ^2^
			Lower	Upper	
Region					
Southern ^a^	Referent				
Eastern ^b^	0.92	0.31	0.47	1.78	0.800
Northern ^c^	1.83	1.18	0.51	6.53	0.348
Breed					
Small East African Zebu	Referent				
Ankole	0.63	0.33	0.22	1.81	0.391
Sex					
Female	Referent				
Male	1.55	0.55	0.76	3.14	0.224
Age of animal at slaughter, years					
0–3.5	Referent				
4–5	5.84	2.20	2.79	12.22	0.001
6–10	2.77	1.78	0.78	9.76	0.113
Body condition score					
3	Referent				
3.5	0.42	0.15	0.21	0.88	0.023
4	0.22	0.18	0.04	1.10	0.064
Gross weight of liver at slaughter, kg					
0–2	Referent				
2.1–4	1.15	0.45	0.52	2.51	0.725
4.1–6	2.96	2.71	0.50	17.80	0.236

^a^ Southern = Amach, Bala, and Boroboro; ^b^ Eastern = Apala, Barr, and Otuke; ^c^ Northern = Achwa, Agweng, Alito, Barlonyo, Mocwari, and Ngetta. ^2^ *p* < 0.05.

## Data Availability

The data presented in this study are available on request from the corresponding author.

## Supplementary Materials

The following are available online at https://www.mdpi.com/2076-2615/11/3/681/s1, Table S1: The financial losses associated with liver condemnation from 142 cows due to fascioliasis in cattle slaughtered at Lira municipality abattoir in Northern Uganda over 14 days.
