# Supplementary material for: Prevalence of Fascioliasis and Associated Economic Losses in Cattle Slaughtered at Lira Municipality Abattoir in Northern Uganda
Source: Animals (Basel). 2021 Mar 4;11(3):681. doi: 10.3390/ani11030681 (PMC7999142; doi:10.3390/ani11030681)
Supplement: Supplementary file 1 [file animals-11-00681-s001.pdf]

animals MDPI

Supplementary Table 1. The financial losses associated with liver condemnation from 142 cows due to fascioliasis in cattle slaughtered at Lira municipality abattoir in Northern Uganda over 14 days.

Kilogram of liver trimmed off	Frequency (n)	Unit monetary loss (UGX)[a]	Total loss (UGX)
0	75	0	0
0.3	6	3,000	18,000
0.4	8	4,000	32,000
0.5	25	5,000	125,000
0.6	12	6,000	72,000
0.7	11	7,000	77,000
0.8	8	8,000	64,000
0.9	11	9,000	99,000
1.0	13	10,000	130,000
1.1	5	11,000	55,000
1.2	3	12,000	36,000
1.3	6	13,000	78,000
1.4	2	14,000	28,000
1.5	11	15,000	165,000
1.6	3	16,000	48,000
1.7	2	17,000	34,000
1.8	1	18,000	18,000
1.9	3	19,000	57,000
2.1	1	21,000	21,000
2.3	1	23,000	23,000
2.4	1	24,000	24,000
2.5	1	25,000	25,000
2.8	2	28,000	56,000
3.0	2	30,000	60,000
3.1	2	31,000	62,000
5.0	1	50,000	50,000
Estimated financial loss			1,457,000

[a]UGX= Ugandan Shillings (one UGX= 0.00027 USD)
